# The transcriptional response to tumorigenic polarity loss in Drosophila

**DOI:** 10.7554/eLife.03189

**Published:** 2015-02-26

**Authors:** Brandon D Bunker, Tittu T Nellimoottil, Ryan M Boileau, Anne K Classen, David Bilder

**Affiliations:** 1 Department of Molecular and Cell Biology, University of California, Berkeley, Berkeley, United States; 2 University of Southern California, Department of Biological Sciences, Los Angeles, United States; The Samuel Lunenfeld Research Institute, Canada

**Keywords:** tumor, cancer, epithelial cells, transcriptome, polarity, *D. melanogaster*

## Abstract

Loss of polarity correlates with progression of epithelial cancers, but how plasma membrane misorganization drives oncogenic transcriptional events remains unclear. The polarity regulators of the *Drosophila* Scribble (Scrib) module are potent tumor suppressors and provide a model for mechanistic investigation. RNA profiling of Scrib mutant tumors reveals multiple signatures of neoplasia, including altered metabolism and dedifferentiation. Prominent among these is upregulation of cytokine-like Unpaired (Upd) ligands, which drive tumor overgrowth. We identified a polarity-responsive enhancer in *upd3*, which is activated in a coincident manner by both JNK-dependent Fos and aPKC-mediated Yki transcription. This enhancer, and Scrib mutant overgrowth in general, are also sensitive to activity of the Polycomb Group (PcG), suggesting that PcG attenuation upon polarity loss potentiates select targets for activation by JNK and Yki. Our results link epithelial organization to signaling and epigenetic regulators that control tissue repair programs, and provide insight into why epithelial polarity is tumor-suppressive.

**DOI:** http://dx.doi.org/10.7554/eLife.03189.001

## eLife digest

The cavities and organs within our body are lined with epithelial cells, which connect to each other to form continuous barriers. These cells have a highly polarized structure in which different components are found at the top and bottom of cells. In the fruit fly and most other animals, three genes known as the Scribble module control the polarity of epithelial cells. If these genes are faulty, the cells lose their polarity, break the epithelial barrier, and grow rapidly to form a tumor. Most malignant tumors that form from epithelial cells have lost normal cell polarity, so understanding how the organization and growth of epithelial cells are linked is a critical question.

It is not clear how the loss of cell polarity can drive tumor formation. Here, Bunker et al. used a technique called RNA sequencing to study the expression of genes in tumor cells that have mutations in the Scribble module. Hundreds of genes in the tumor cells had different levels of expression from the levels seen in normal fly cells. One of these is a gene called *upd3*, which was expressed much more highly in tumor cells than in normal cells. This gene activates a signaling pathway—called the JAK/STAT pathway—that promotes cell growth and division in many animals. Bunker et al. found that experimentally lowering the activity of the JAK/STAT pathway reduced the growth of the tumor cells that had lost normal polarity.

Further experiments show that disrupting the layer of epithelial cells activates two other signaling pathways that work together to switch on the *upd3* gene when cell polarity is lost. Proteins belonging to the Polycomb Group also control the expression of *upd3* and other genes involved in cell growth by altering how genetic material is packaged in cells.

The similarities between this response and the response to tissue damage suggest that the loss of polarity drives tumor formation through an unstoppable wound-healing reaction. Therefore, Bunker et al.'s findings link the formation of epithelial tumors to the signaling pathways that control the repair of damaged tissues.

**DOI:** http://dx.doi.org/10.7554/eLife.03189.002

## Introduction

The diagnosis of carcinomas–malignant tumors of epithelial origin—has long involved evaluating tissue architecture. Pronounced disorganization of biopsied epithelia is well-established to correlate with tumor malignancy and lethality. However, whether there exists a causative relationship between epithelial organization and tumor progression, as well as what the underlying mechanism might be, has been mysterious. Recent years have shed important light on the former question, identifying contexts where altered activity of proteins that regulate epithelial cell polarity can promote oncogenic phenotypes. For instance, the apical determinant atypical protein kinase C (aPKC) is amplified and over-expressed in multiple cancers (Huang and Muthuswamy, 2010; Parker et al., 2014), while basolateral regulators are altered in several tumor types and degraded by viral oncoproteins (Huang and Muthuswamy, 2010; Elsum et al., 2012); cancer stem cell activity may also be promoted by transition from an epithelial state (Martin-Belmonte and Perez-Moreno, 2011; Scheel and Weinberg, 2012). Mouse models continue to support key roles for polarity regulators in cancer progression (Pearson et al., 2011; Muthuswamy and Xue, 2012; Elsum et al., 2014; Feigin et al., 2014), but the mechanisms linking epithelial organization to tissue homeostasis, as well as the cellular targets that promote oncogenic growth upon polarity loss, remain unclear.

Early evidence for causative links emerged from *Drosophila*, where mutations in single polarity-regulating genes can induce dramatic tumorous growths. These polarity regulators–*scribble* (*scrib*), *discs-large* (*dlg*), and *lethal giant larvae* (*lgl*)*-* cooperatively distinguish the basolateral domain from the apical by antagonizing aPKC activity (St Johnston and Ahringer, 2010; Tepass, 2012). This conserved ‘Scrib module’ functions in both vertebrates and invertebrates, not only in epithelia but also other polarized cell types. Conservation of these and other core polarity regulators allows *Drosophila* to be used as a model to study the coupling between epithelial architecture and growth control.

When Scrib module function is lost from fly epithelia, mutant cells round up and become multilayered. In the imaginal discs, epithelial organs which normally have a precise intrinsic size-control mechanism, mutant tissue continuously proliferates to more than five times the WT cell number before it kills the animal. Small portions of the tumorous mass, when transplanted into adults, continue to grow uncontrollably and kill the host; such allografts can be repeated indefinitely. This disorganized, lethal and transplantable growth has been termed ‘neoplastic’, and includes several additional features (Gateff and Schneiderman, 1969; Bilder, 2004). Neoplastic fly tissue is prone to dissemination and degrades basement membrane; in cooperation with oncogenic Ras it can migrate away from its primary site and invade other organs (Pagliarini and Xu, 2003). It is compromised in its differentiation potential, and cannot form adult structures (Gateff and Schneiderman, 1969). It can be recognized by the host innate immune system, whose cellular activities impede its growth (Pastor-Pareja et al., 2008; Cordero et al., 2010). Finally, it produces long-range signals that induce detrimental responses in fly hosts, including cachexia-like tissue wasting (Figueroa-Clarevega and Bilder, 2015). This suite of phenotypes, which echo those found in mammalian malignancies, suggest that elucidating mechanisms linking epithelial organization to tumor suppression in flies may provide novel insight into human cancer as well.

What are the genes that induce the multiple aspects of the neoplastic phenotype, and how does loss of a single polarity regulator at the plasma membrane lead to their nuclear misregulation? Here we define the global transcriptional changes associated with tumorigenic epithelial disorganization. By focusing on a single polarity-regulated enhancer of a gene involved in overgrowth, we then untangle signaling, transcription factor, and epigenetic activities that mediate activation upon polarity loss. Our results suggest that epithelia monitor their integrity via a coincidence detection mechanism, and respond to its loss by activating a damage-responsive gene expression program that cannot be turned off in mispolarized tumors.

## Results

### Polarity disruption drives oncogenic transcriptional changes

**Figure 1.**
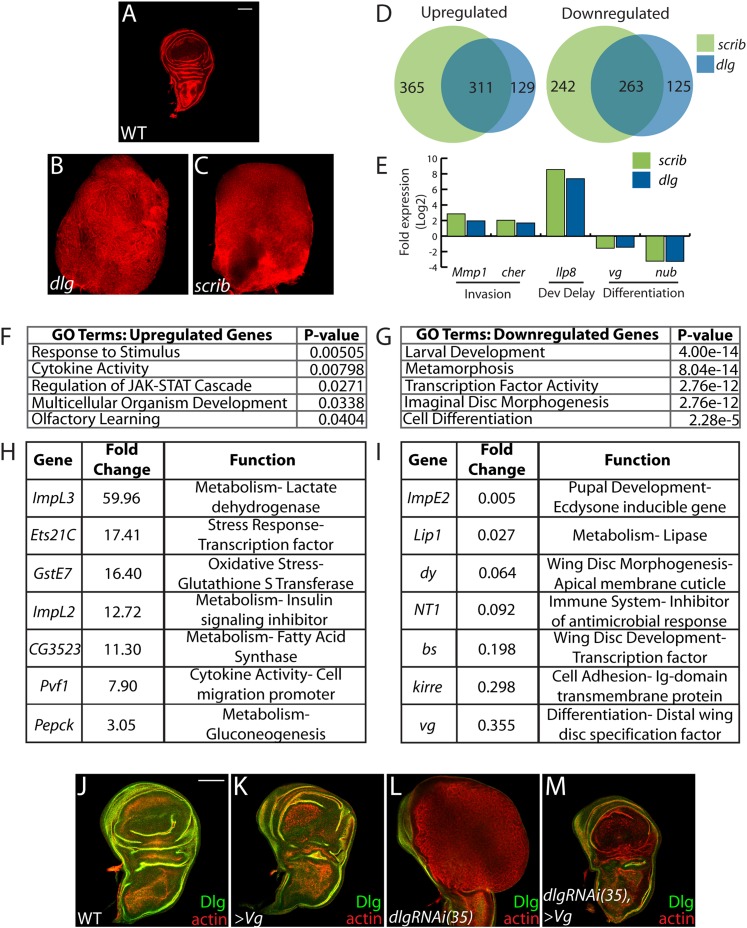
Transcriptome analysis of neoplastic tumors. (**A**–**C**) F-actin staining reveals dramatic overgrowth and architecture defects of neoplastic *dlg* and *scrib* wing discs relative to WT. (**D**) Overlap of genes upregulated (left) or downregulated (right) in *scrib* and *dlg* tissues. (**E**) Genes previously implicated in neoplastic characteristics are differentially expressed. (**F** and **G**) Functional categories enriched in the upregulated and downregulated genes include markers of stress response and JAK/STAT pathway activation, and de-differentiation respectively. Selected overexpressed (**H**) and underexpressed (**I**) genes are shown. (**J**–**M**) Overexpression of *Vg* suppresses *dlgRNAi*-driven overgrowth and architecture defects. Dlg staining (green) demonstrates survival of Dlg-depleted wing cells. Scale bars: 100 μm. **DOI:** http://dx.doi.org/10.7554/eLife.03189.003

The many malignant-like phenotypes observed upon loss of a single polarity regulator must be driven by altered gene expression. To identify such genes, we carried out RNA-Seq analysis of WT and mutant wing imaginal discs. We focused on changes common to neoplasm by sequencing cDNA libraries generated from both *scrib* and *dlg* tumors, which phenocopy each other (Figure 1A–C) (Bilder et al., 2000). Analysis revealed 574 genes misregulated at least twofold in both mutant tissues (FDR <5%), with 311 and 263 up- and downregulated respectively (Figure 1D and Suplementary files 1–2). Differentially expressed genes include several previously identified neoplastic effectors, such as the pro-invasion factors *Matrix metalloprotease 1* (*Mmp1*) and *cheerio* (*cher*) as well as the pupation regulator *insulin-like peptide 8* (*Ilp8*) (Uhlirova and Bohmann, 2006; Colombani et al., 2012; Garelli et al., 2012; Külshammer and Uhlirova, 2013) (Figure 1E). qRT-PCR analysis of these and other genes shows close agreement with RNA-Seq data (R^2^ = 0.8844). The transcriptome dataset therefore accurately captures the expression profile of neoplastic tissues, and contains genes that promote tumorigenesis upon polarity loss.

**Figure 1—figure supplement 1.**
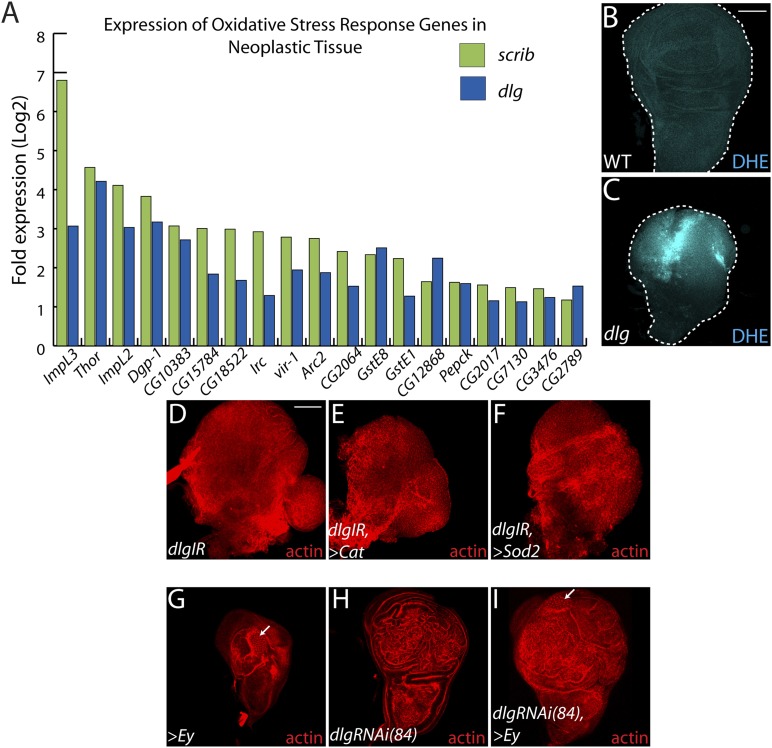
Decreasing oxidative stress or reexpressing *eyelesss* does not suppress neoplasia. (**A**) 19 genes activated in response to oxidative stress are significantly upregulated upon polarity loss. (**B**–**C**) Loss of *dlg* leads to higher superoxide levels, as evidenced by increased DHE staining, relative to WT. (**D**–**F**) Expression of the anti-oxidant enzymes *Cat* or *Sod2* has no effect on *dlgRNAi*-mediated overgrowth. (**G**–**I**) Ectopic expression of *eyeless* (*eye*), a master regulator of eye differentiation, induces photoreceptor formation in a small portion of wild-type and *dlgRNAi(84)*-expressing discs (arrows), and is unable to block overgrowth upon polarity disruption. Scale bars: 100 μm. **DOI:** http://dx.doi.org/10.7554/eLife.03189.004

Amongst upregulated genes, Gene Ontology (GO) highlights factors involved in Response to Stimulus (Figure 1F,H). Several in this category are immune-related factors, and may be due to the recruitment of hemocytes to neoplastic tumors (Lebestky et al., 2000; Pastor-Pareja et al., 2008; Cordero et al., 2010). Others, including *Glutathione S transferase E1* (*GstE1*) and the chaperone *CG7130*, are regulated by oxidative stress, and overall 19 polarity-sensitive targets are also elevated in hyperoxic conditions (Figure 1—figure supplement 1A) (Landis et al., 2004). Dihydroethidium (DHE), a fluorescent probe for superoxide anions, readily demonstrated elevation upon depletion of *dlg* (Figure 1—figure supplement 1B–C). Co-overexpression of *Catalase*, *Superoxide dismutase 2*, or rat *Glutathione Peroxidase 1*, which suppress other *Drosophila* ROS dependent phenotypes (Owusu-Ansah and Banerjee, 2009; Ohsawa et al., 2012; Lim et al., 2014) failed to alter the neoplastic phenotype induced by *dlg* knockdown (Figure 1—figure supplement 1D–F), although we were unable to detect a consistent reduction of DHE in these contexts. Several metabolic regulators are also misexpressed in polarity-deficient tissues, including *Drosophila Lactate Dehydrogenase* (*ImpL3*), which contributes to a Warburg-like metabolic shift in human tumors (Cairns et al., 2011); however, *ImpL3* knockdown also did not obviously alter neoplastic growth (data not shown).

Primary GO categories among downregulated genes likely reflect the failure of neoplastic tumors to differentiate (Figure 1E,G,I). We investigated the functional role by ectopically expressing fate-specifying transcription factors in *dlg*-depleted tissue. Strikingly, co-expression of *vestigial* (*vg*), a distal wing pouch selector that is downregulated in mutant discs, suppressed overgrowth and architecture defects (Figure 1J–M). Though *vg* overexpression eliminates polarity-deficient clones through apoptosis (Khan et al., 2013), we recovered an intact wing pouch consisting of *dlgRNAi/vg* co-expressing cells (Figure 1M). We also tested ectopic expression of an eye-specifying transcription factor in wing and eye tissue. *eyeless* was incapable of suppressing *dlg* knockdown in either context, but was also incapable of inducing broad photoreceptor differentiation in WT or *dlg-*depleted tissue (Figure 1—figure supplement 1G–I, data not shown). Together, these data suggest that restoring expression of differentiation-promoting transcription factors can, in some contexts, block neoplastic transformation.

### JAK-STAT ligand transcription promotes neoplastic overgrowth

**Figure 2.**
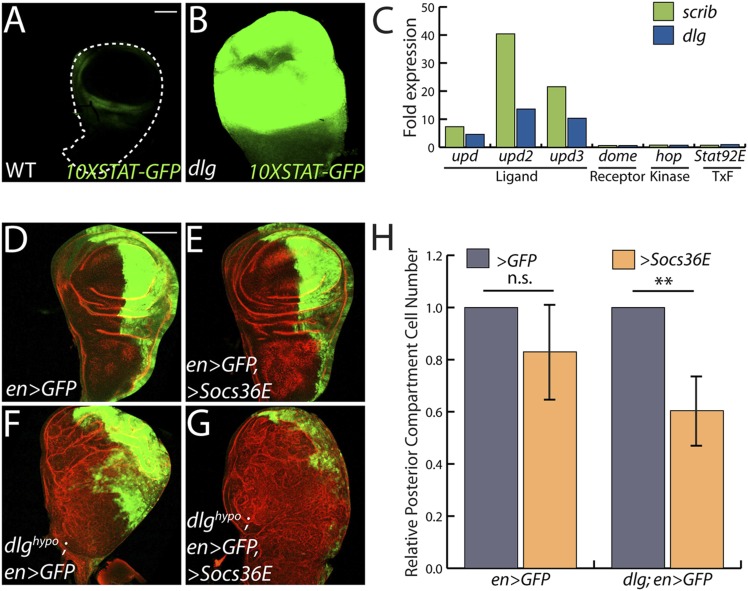
JAK/STAT activation drives overgrowth upon polarity loss. (**A** and **B**) A JAK/STAT pathway reporter (green) is highly elevated throughout *dlg* as compared to WT discs, indicating strong pathway activation. (**C**) The ligand-encoding *upd* genes, but not other JAK/STAT pathway components, are transcriptionally upregulated in neoplastic tissues. (**D**–**G**) Reduction of JAK/STAT pathway activity via SOCS36E overexpression has no significant effect on WT growth, but suppresses overgrowth of *dlg*^*hypo*^ tissue. Actin (red) highlights cell outlines, while GFP (green) marks the *engrailed-*expressing domain. FACS-based quantification is shown in **H** (**p < 0.001). Scale bars: 100 μm. **DOI:** http://dx.doi.org/10.7554/eLife.03189.005

The only cell signaling pathway among the top GO categories is the JAK/STAT cascade. Upregulated genes include STAT targets such as *chinmo* and *Socs36E*, and a JAK/STAT activity reporter is strongly expressed in *dlg* and *scrib* discs (Figure 2A–B) (Wu et al., 2010). Remarkably, each of the three *unpaired* (*upd*) genes, which encode the ligands for the JAK/STAT pathway, were transcriptionally elevated between ∼3- and ∼50-fold, while genes encoding other signal transduction components were unaltered (Figure 2C). To assess a functional role, we used *engrailed-GAL4* to express *Socs36E*, a negative regulator of JAK/STAT intracellular signaling (Callus and Mathey-Prevot, 2002), in the posterior compartment of wing discs carrying a hypomorphic allele of *dlg*, and then counted cell numbers on a cell sorter. Strikingly, *Socs36E* decreased proliferation of *dlg*^*hypo*^ cells by 40%, while having no significant effect on growth or viability of WT discs (Figure 2D–H). Expression of *Socs36E* or a dominant-negative form of the JAK-STAT receptor *Domeless* (*Dome*^*DN*^) also suppressed the growth of *scrib-*depleted discs (Figure 4—figure supplement 3A–C). We therefore conclude that in imaginal discs, as in *RasV12*-expressing clones (Wu et al., 2010), the Scrib module regulates JAK-STAT ligand expression to suppress tissue overgrowth.

**Figure 2—figure supplement 1.**
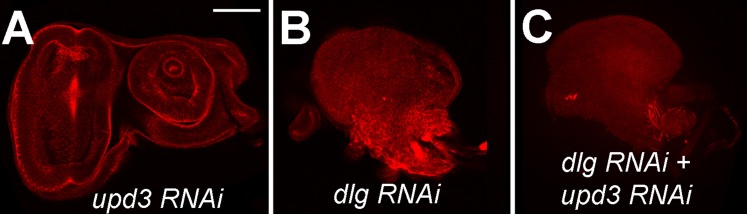
*upd3* knockdown is not sufficient to prevent neoplastic tumors. Eye imaginal discs expressing *upd3 RNAi* alone (**A**), *dlg RNAi* alone (**B**), and *dlg RNAi* + *upd3 RNAi* (**C**). Scale bar: 50 μm. **DOI:** http://dx.doi.org/10.7554/eLife.03189.006

### Isolation of a polarity-responsive enhancer in *upd3*

**Figure 3.**
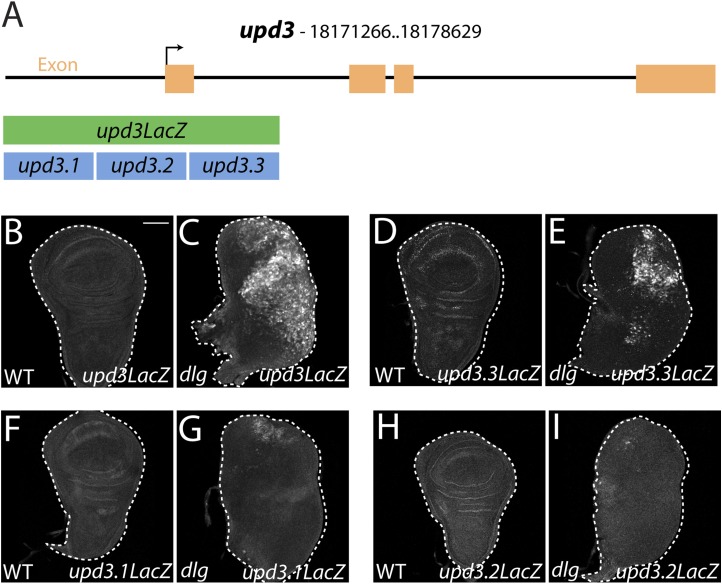
Identification of a polarity-responsive enhancer in *upd3.* (**A**) Schematic of *upd3* reporter constructs in relation to the corresponding genomic region. (**B** and **C**) 3 kb *upd3LacZ* is not expressed in WT, but is upregulated in *dlg* discs. (**D** and **E**) *upd3.3LacZ* sub-fragment is also silent in WT, but is upregulated in *dlg* like *upd3LacZ*. (**F**–**I**) Other sub-fragments are not significantly expressed in either WT or *dlg*. Scale bar: 100 μm. **DOI:** http://dx.doi.org/10.7554/eLife.03189.007

**Figure 4.**
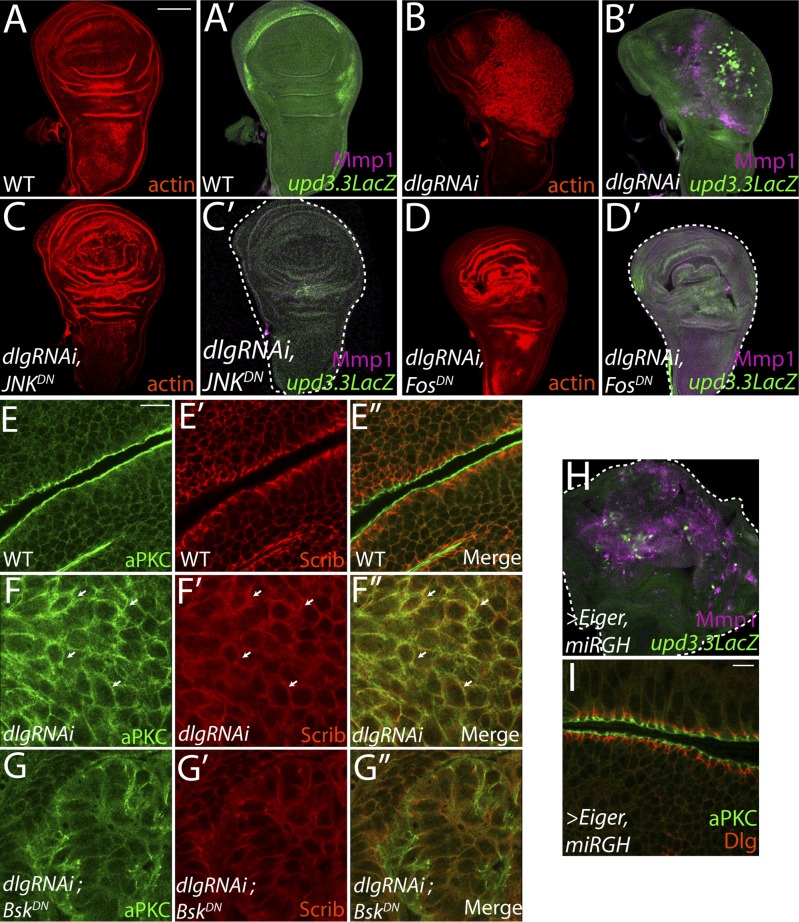
JNK-Dependent transcription is necessary for overgrowth and *upd3.3* activation upon polarity loss. WT wing discs (**A**) do not express either the JNK target Mmp1 or *upd3.3LacZ* (**A′**). Expression of *dlgRNAi* promotes overgrowth and disorganization (**B**), as well as Mmp1 and *upd3.3LacZ* upregulation (**B′**). Inhibiting AP-1 transcription with either *JNK*^*DN*^ or *Fos*^*DN*^ restores normal disc size and architecture (**C** and **D**), and abrogates Mmp1 and *upd3.3LacZ* expression (**C′** and **D′**). WT discs segregate apical aPKC and basolateral Scrib (**E**). *dlgRNAi* expression leads to apical domain expansion and co-localization of aPKC and Scrib (**F**, arrowheads). Co-expressing *JNK*^*DN*^ and *dlgRNAi* restores the separation of aPKC and Scrib (**G**). Activation of JNK is sufficient, when apoptosis is blocked with *miRGH*, to drive *upd3.3LacZ*, Mmp1 and overgrowth but not to alter polarity (**H** and **I**). Scale bars: **A**–**D**, **H**: 100 μm, **E**–**G**, **I**: 10 μm. **DOI:** http://dx.doi.org/10.7554/eLife.03189.010

To elucidate links between polarity and transcriptional control of growth, we focused on a single mitogenic gene: *upd3*. We cloned 3 kilobases (kb) of genomic DNA surrounding the *upd3* ATG into a *lacZ* reporter (‘*upd3lacZ*’) and found that this reporter was not expressed in WT discs. However, like the overlapping *upd3 > GFP* reporter, it was distinctly upregulated in neoplastic discs (Figure 3A–C) (Pastor-Pareja et al., 2008). We then identified a minimal polarity-responsive region within this enhancer, using fragments previously analyzed in the adult gut (Jiang et al., 2011). Although reporters including *upd3.1LacZ*, which is activated by perturbations in the gut epithelium, remain silent, a 1-kb element within the first intron (*upd3.3LacZ*) was expressed in a patchy manner throughout *dlg* discs (Figure 3D–I). Expression of *upd3.3lacZ*, like that of *upd3lacZ*, was in cells of the disc proper, not in the peripodium or hemocytes (Figure 3—figure supplement 1A–B); this patchy expression resembled that seen with several other upregulated neoplastic effectors, (Figure 4B′, Figure 3—figure supplement 1C–H). *Upd3.3LacZ* was similarly activated in *scrib* discs, demonstrating that this enhancer is generally responsive to disruption of epithelial polarity (Figure 4—figure supplement 3E) and identifying a polarity-sensitive *cis*-regulatory region.

**Figure 3—figure supplement 1.**
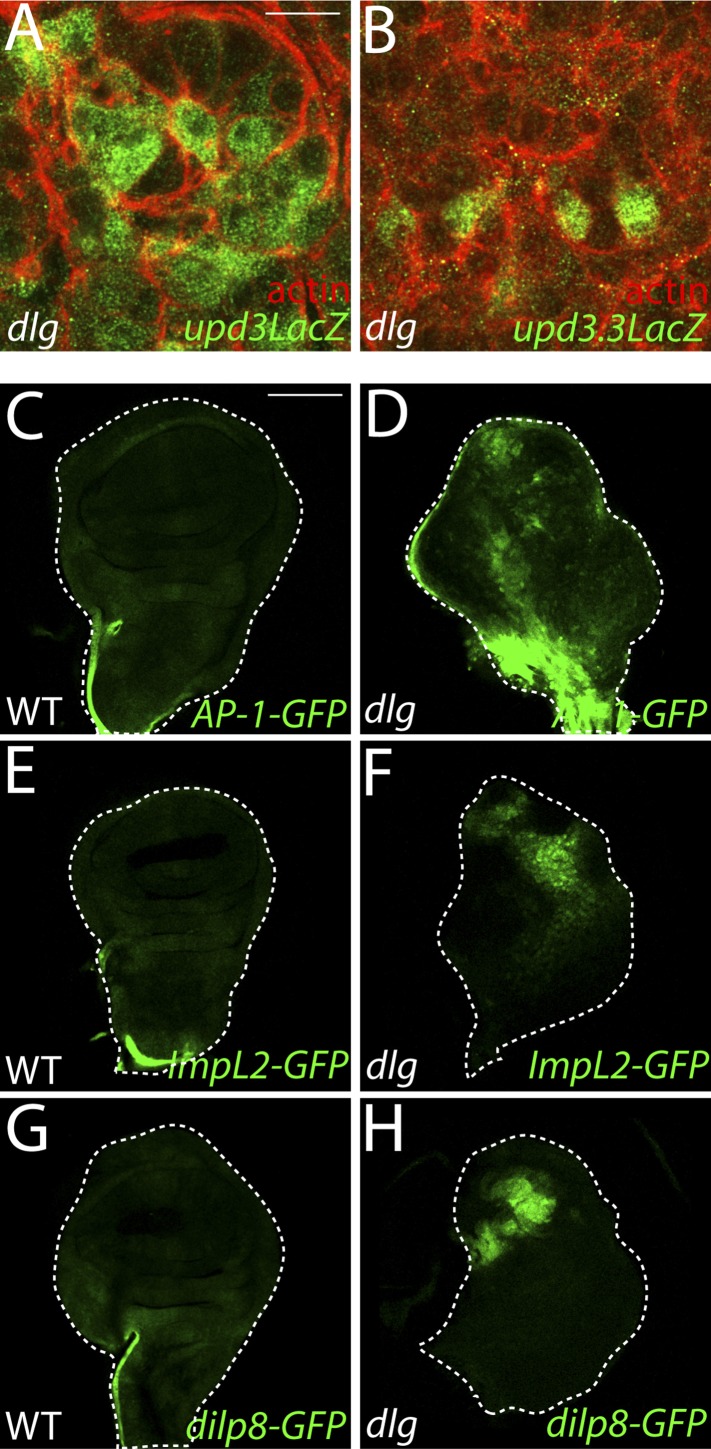
Imaginal expression of polarity-responsive target genes in neoplasia. (**A**–**B**) The *upd3LacZ* and *upd3.3LacZ* reporters are expressed primarily in the disc proper, and not the hemocytes or the peripodial membrane. (**C**–**H**) The JNK pathway reporter *AP-1-GFP*, and transcriptional reporters for the polarity-sensitive targets *ImpL2* and *dilp8* are relatively silent in WT tissue, but active in a patchy pattern in *dlg* discs. Scale bars: **A**–**B**: 10 μm, **C**–**H**: 100 μm. **DOI:** http://dx.doi.org/10.7554/eLife.03189.008

**Figure 3—figure supplement 2.**
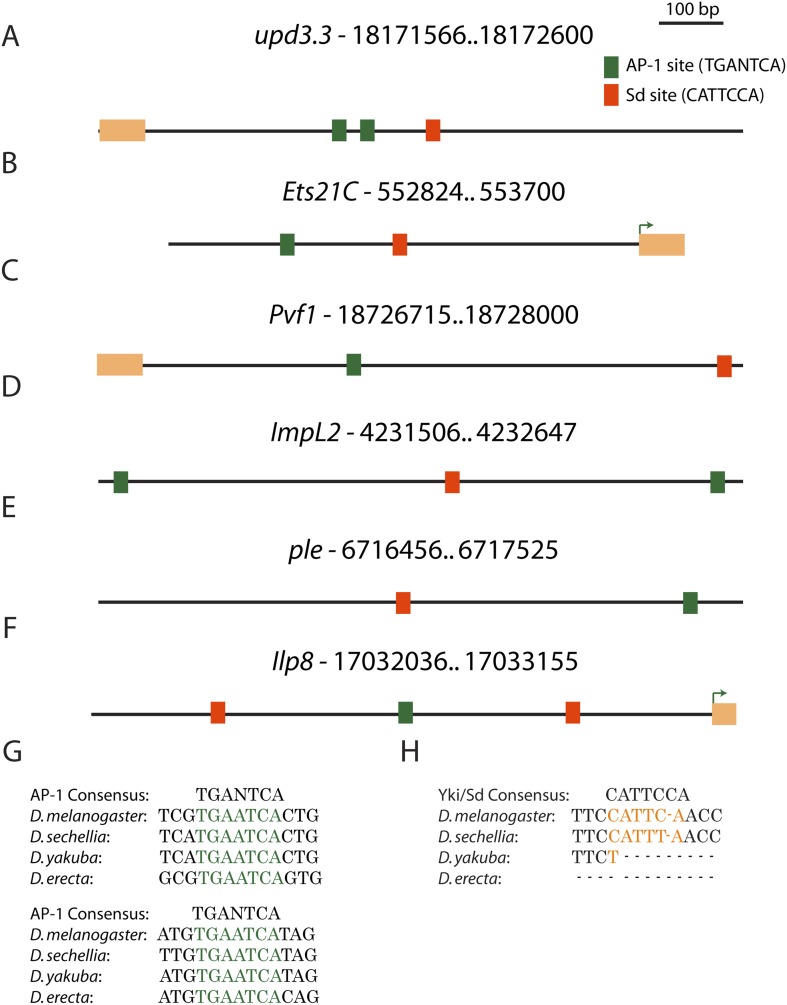
Conserved AP-1 and Sd binding sites in genes upregulated in neoplasia. (**A**) The *upd3.3* enhancer contains two evolutionarily conserved (between *D. melanogaster*, *D. yakuba* and *D. erecta*) AP-1 binding sites (green boxes), and one semi-conserved Sd binding site (red box). Conserved AP-1 and Sd binding sites are also evident in several neoplasia-induced genes that are also upregulated during wounding, including *Ets21C* (**B**), *Pvf1* (**C**), *ImpL2* (**D**), *ple* (**E**), and *Ilp8* (**F**). Exons are denoted in orange and green arrows in *Ets21C* and *Ilp8* mark the transcription start site. (**G** and **H**) The conservation of the AP-1 and Sd binding sites in *upd3.3* is shown. **DOI:** http://dx.doi.org/10.7554/eLife.03189.009

**Figure 4—figure supplement 1.**
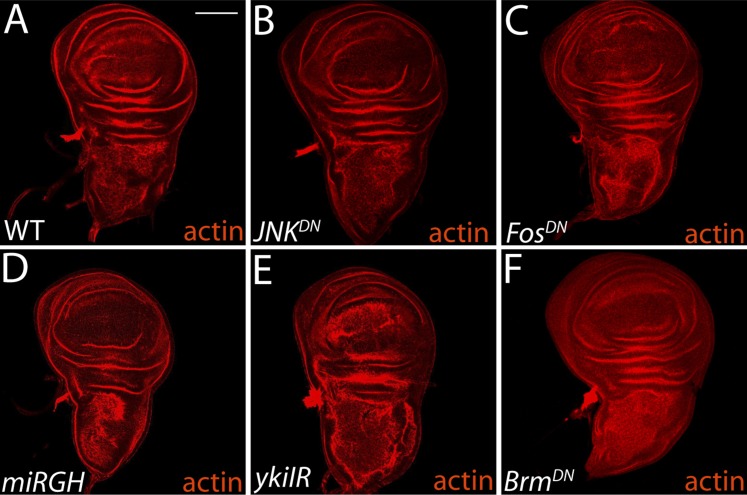
Inhibitor constructs do not significantly affect WT tissue growth and viability. (**A**–**B**) Blocking JNK activity with *JNK*^*DN*^ or *Fos*^*DN*^ (**C**) has no effect on normal growth or tissue architecture, relative to wild-type. Expression of *miRGH* does not affect normal tissue architecture or growth (**D**). Knockdown of Yki promotes mild architecture defects (**E**), while *Brm*^*DN*^ expression has no phenotype (**F**). For all panels, transgenes were expressed in the dorsal wing pouch with the *ms1096-GAL4* driver. Scale bar: 100 μm. **DOI:** http://dx.doi.org/10.7554/eLife.03189.011

**Figure 4—figure supplement 2.**
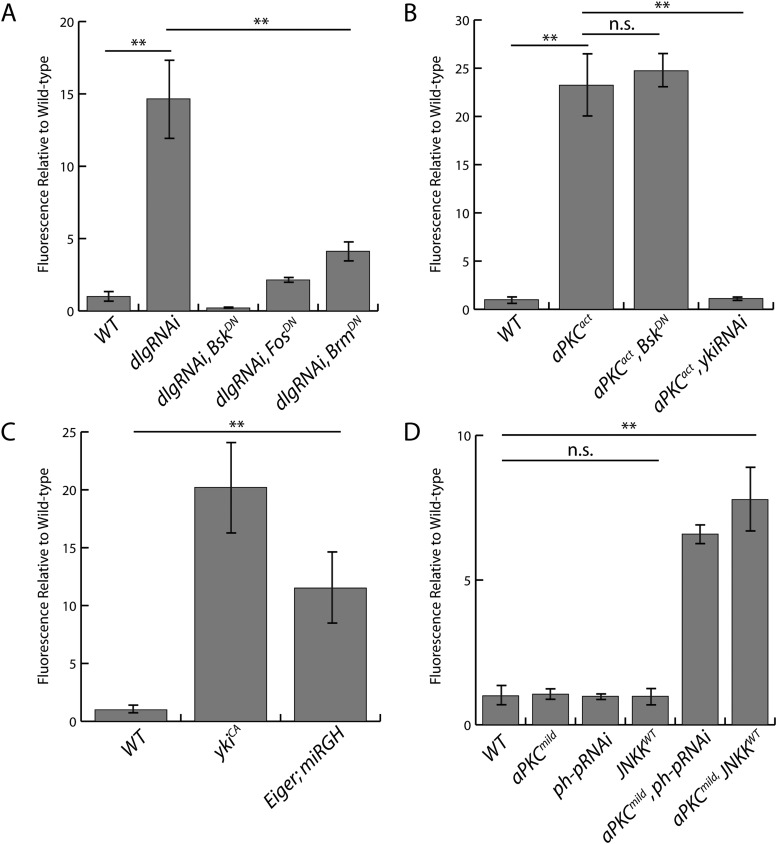
Quantification of *upd3.3LacZ* staining. (**A**) Expression of *dlgRNAi* increases *upd3.3LacZ* fluorescence, which is suppressed by blocking JNK or Trx activity. (**B**) Expression of *aPKC*^*act*^ stimulates *upd3.3LacZ* in a JNK-independent, but Yki-dependent manner. (**C**). Hyperactivation of Yki or JNK activity upregulates *upd3.3LacZ* expression. (**D**) Alone, expression of *aPKC*^*mild*^, *ph-pRNAi*, or *JNKK*^*WT*^ does not activate *upd3.3LacZ*; however, co-expression of *aPKC*^*mild*^ with *ph-pRNAi* or *JNKK*^*WT*^ drives *upd3.3LacZ*. (**p < 0.001; n.s. = not significant). **DOI:** http://dx.doi.org/10.7554/eLife.03189.012

**Figure 4—figure supplement 3.**
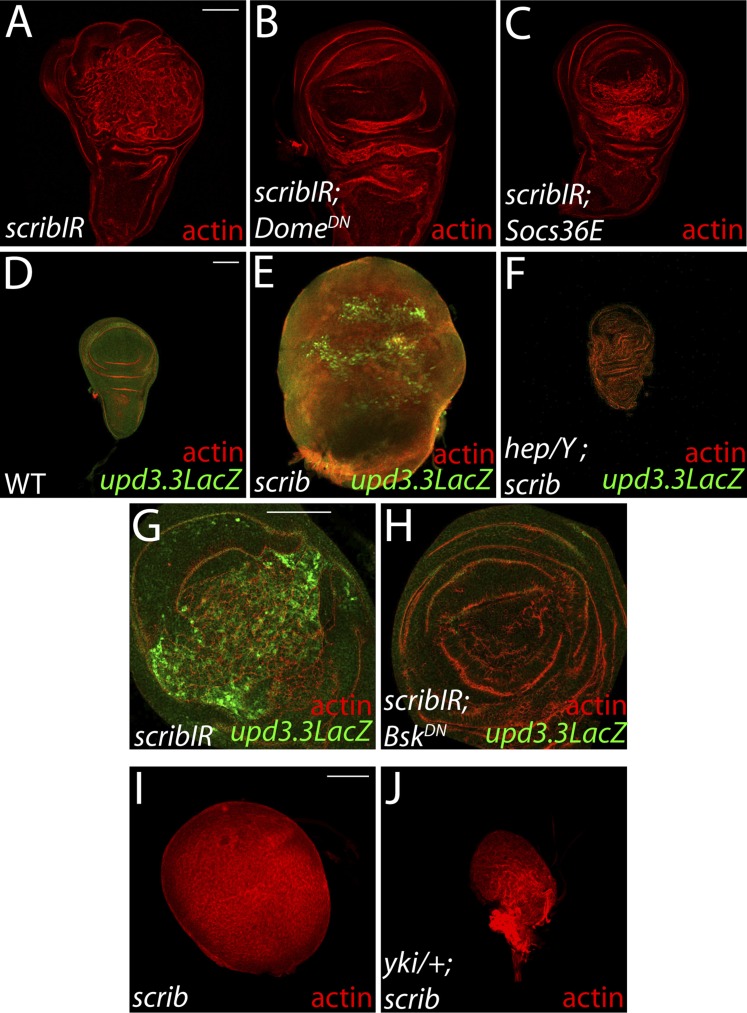
Neoplasia induced by *scrib* loss is also dependent on JAK-STAT, JNK, and Yki pathway activity. (**A**–**C**) Reducing JAK-STAT activity with *Dome*^*DN*^ or *Socs36E* attenuates *scribIR*-mediated overgrowth. (**D**–**H**) Blocking JNK pathway activation by depletion of the JNK kinase *hep* or overexpression of *JNK*^*DN*^ suppresses the overproliferation, architecture defects and *upd3.3LacZ* activation induced by *scrib* loss. (**I** and **J**) Yki is necessary for neoplastic overgrowth of *scrib* tissue. Scale bar: 100 μm. **DOI:** http://dx.doi.org/10.7554/eLife.03189.013

### JNK-mediated transcription drives *upd3.3* expression upon polarity loss

We next sought to identify molecular pathways linking epithelial polarity to *upd3* expression. Motif scanning of the *upd3.3* enhancer detected two evolutionarily-conserved binding sites for AP-1, the Jun kinase (JNK) pathway transcription factor (Figure 3—figure supplement 2A,G). We tested whether JNK signaling is required for *upd3.3LacZ* activation. Expression of a dominant-negative form of *Drosophila* JNK (Flybase: *Basket*), (*JNK*^*DN*^) has been shown to block neoplastic overgrowth, as well as polarity and architecture defects (Figure 4A–C,E–G; Figure 4—figure supplement 1A–B) (Robinson and Moberg, 2011; Sun and Irvine, 2011). Notably, *JNK*^*DN*^ also completely abrogated *dlgRNAi*-induced *upd3.3LacZ* expression (Figure 4B′,C′; Figure 4—figure supplement 2A), as well as that induced by *scribRNAi* (Figure 4—figure supplement 3G–H). Mutation of the JNK kinase, *hemipterous* (*hep*) also prevented *upd3.3LacZ* levels in *scrib* tissue (Figure 4—figure supplement 3D–F), confirming that canonical JNK signaling acts downstream of polarity disruption to regulate *upd3*.

The mechanism by which JNK promotes neoplasia is unclear. Phosphorylation of Ajuba LIM protein (Jub) has been proposed to be key (Sun and Irvine, 2013); however, the presence of AP-1 binding sites within *upd3.3* suggests a direct transcription-mediated mechanism. To test the latter mechanism, we assayed discs co-expressing *dlgRNAi* and *fos*^*DN*^, which prevents activity of the AP-1 transcription factor (Ciapponi et al., 2001). Strikingly, *fos*^*DN*^ fully phenocopied the effects of *JNK*^*DN*^: it prevented both *upd3.3LacZ* expression and *dlgRNAi-*mediated neoplasia (Figure 4D; Figure 4—figure supplement 1C; Figure 4—figure supplement 2A). Taken together, these experiments demonstrate that maintenance of epithelial polarity prevents transcription of oncogenic JNK-dependent target genes.

Given that elevated JNK signaling is necessary for *upd3.3LacZ* expression and neoplastic overgrowth, is it sufficient? Ectopic JNK activity in WT tissue leads to apoptosis (Igaki et al., 2002), so we co-expressed the JNK-activating ligand Eiger with a microRNA targeting the pro-apoptotic genes *reaper*, *grim*, and *head involution defective* (*miRGH*) to block both cell death and caspase activation (Siegrist et al., 2010). In this context, JNK activation alone induced *upd3.3LacZ* (Figure 4H; Figure 4—figure supplement 1D; Figure 4—figure supplement 2C) and increased tissue size (Pérez-Garijo et al., 2009). However, *upd3.3LacZ* induction was low compared to the canonical JNK target Mmp1, while *dlg* knockdown activated both comparably (Figure 4B′,H). Further, apical and basolateral proteins remained properly localized, indicating that JNK activation alone does not disrupt polarity (Figure 4I) (Sun and Irvine, 2011). Therefore, JNK signaling is sufficient for partial *upd3.3* activation and overgrowth, but it is unable to induce full neoplasia.

### aPKC can regulate polarity-responsive transcription, independently of JNK

**Figure 5.**
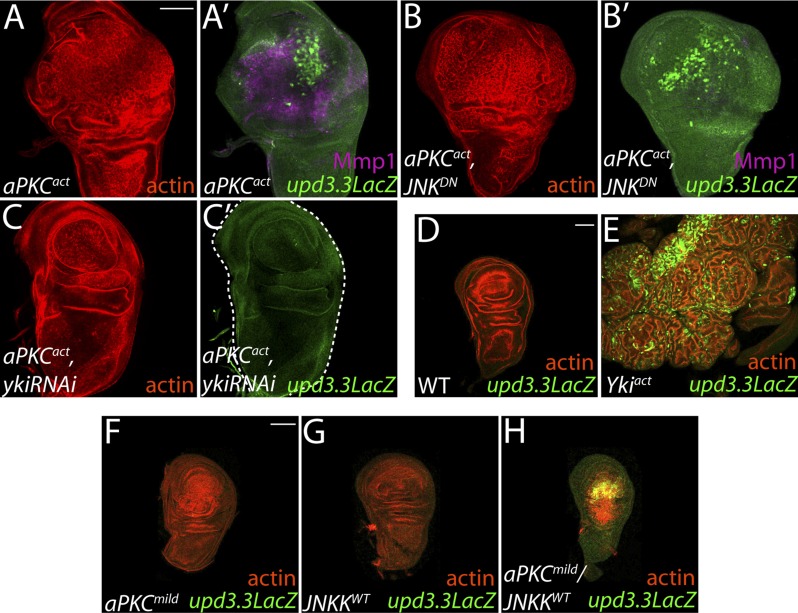
aPKC activity drives *upd3.3LacZ* activation in a *yki*-dependent manner. (**A**) Expression of constitutively active aPKC (*aPKC*^*act*^) induces *upd3.3LacZ* and Mmp1 upregulation and neoplasia. (**B**) Expressing *JNK*^*DN*^ suppresses Mmp1, but does not prevent *aPKC*^*act*^-mediated *upd3.3LacZ* activation or overgrowth. (**C**) Knockdown of *yki* blocks *upd3.3LacZ* and overgrowth upon ectopic *aPKC* activity, while constitutively active Yki drives *upd3.3LacZ* expression and tissue overgrowth relative to WT (**D** and **E**). Expression of a mildly-active form of *aPKC* (**F**) or JNK (**G**) alone cannot activate *upd3.3*, but together are sufficient for expression (**H**). Scale bars: 100 μm. **DOI:** http://dx.doi.org/10.7554/eLife.03189.014

The inability of JNK activation to fully recapitulate *dlg* loss suggests that polarity regulators modulate additional factors to prevent *upd3.3* transcription and neoplasm. One candidate is aPKC, which is strongly mislocalized upon loss of Scrib module function but not JNK activation (Figure 4F,I) (Bilder and Perrimon, 2000). We expressed a constitutively active form (*aPKC*^*act*^) that can drive neoplasia and found that it was sufficient to potently trigger *upd3.3LacZ* transcription (Figure 5A; Figure 4—figure supplement 2B). *aPKC*^*act*^ can also activate JNK targets (Figure 5A′), raising the possibility that aPKC regulates *upd3* through JNK. However, inhibiting JNK did not prevent *aPKC*^*act*^-mediated *upd3.3LacZ* activation or overgrowth, while it was effective at preventing expression of Mmp1 (Figure 5B; Figure 4—figure supplement 2B)*.* Similar results were seen when membrane-bound WT aPKC (*aPKC*^*mild*^) was co-expressed with its partner Par-6, demonstrating that the results are not transgene-specific (Figure 5—figure supplement 1) and thus showing that aPKC is capable of stimulating tumorigenic transcription independently of JNK.

**Figure 5—figure supplement 1.**
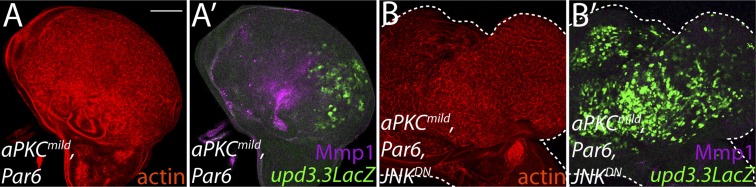
Ectopic aPKC activity drives *upd3.3LacZ* in a JNK-independent manner. (**A**) Expression of *aPKC*^*mild*^ and its partner *Par6* drives *upd3.3LacZ* as well as strong overgrowth and *Mmp1* expression in the wing pouch. (**B**) Co-expression of *JNK*^*DN*^ does not suppress *upd3.3LacZ* activation or overgrowth though Mmp1 upregulation is abrogated. Scale bar: 100 μm. **DOI:** http://dx.doi.org/10.7554/eLife.03189.015

**Figure 5—figure supplement 2.**
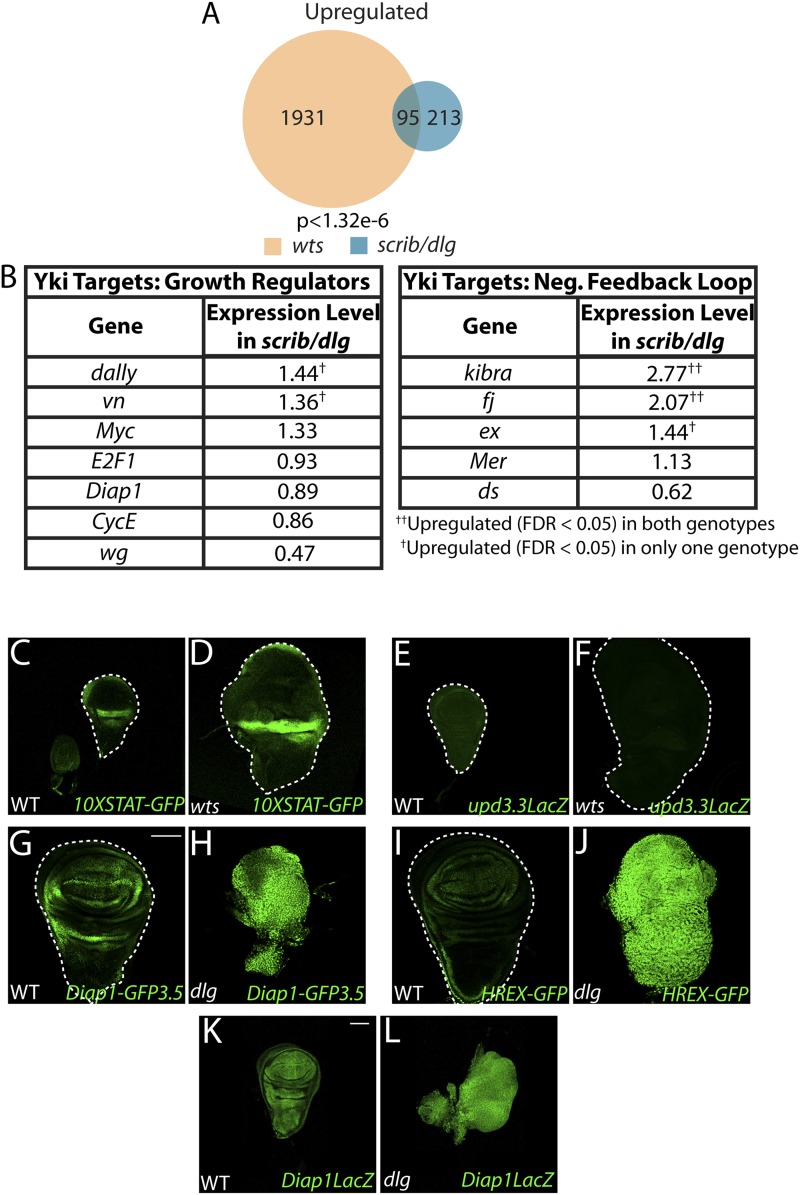
Scrib module and *wts* mutant expression profiles display limited overlap. (**A**) Comparison of the Scrib module and *wts* mutant transcriptomes (see ‘Materials and methods’) reveals a limited degree of overlap. (**B**) Most canonical Yki growth targets are not upregulated in neoplastic tissues. (**C**–**F**) *upd3.3LacZ* and STAT signaling are not upregulated in *wts* discs. The transgenic Hpo pathway reporters *Diap1-GFP3.5* (**G**–**H**) and *HREX-GFP* (**I**–**J**) are strongly upregulated in neoplastic tissue relative to WT; however, *Diap1-LacZ* (**K**–**L**), which is inserted into the endogenous locus, is only slightly increased. Scale bar: 100 μm. **DOI:** http://dx.doi.org/10.7554/eLife.03189.016

**Figure 5—figure supplement 3.**
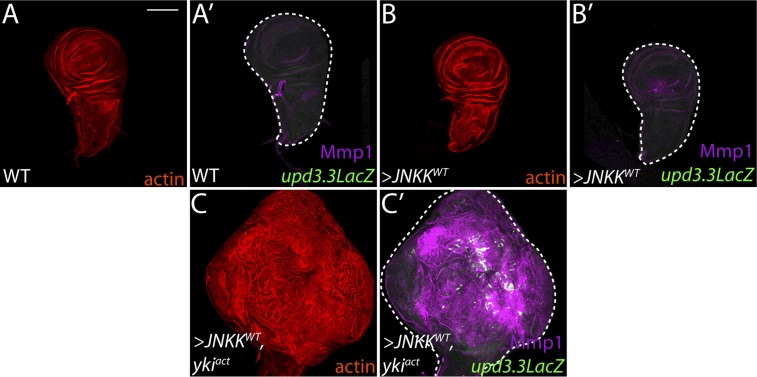
Co-activation of JNK and Yki are not sufficient to drive neoplasia. (**A**–**B**) Ectopic expression of wild-type JNKK causes only slight morphological defects and upregulates Mmp1, but cannot activate *upd3.3LacZ*. (**C**) Co-expression of JNKK and Yki^act^ activates both *upd3.3LacZ* and Mmp1, but does not recapitulate polarity defects or neoplastic-like overgrowth. Scale bar: 100 μm. **DOI:** http://dx.doi.org/10.7554/eLife.03189.017

### aPKC activates polarity-responsive enhancers via Yki

To determine how aPKC activity at the cell cortex regulates transcriptional targets, we returned to our analysis of *upd3.3* sequences. The enhancer contains a partially evolutionarily conserved binding site for Scalloped (Sd), a DNA-binding protein that recruits activated Yorkie (Yki) to target genes (Figure 3—figure supplement 2A,H) (Wu et al., 2008). Intriguingly, conserved Sd and AP-1 binding sites are also found together in ∼1 kb regulatory regions of other upregulated genes (Figure 3—figure supplement 2B–F). To determine if Yki acts downstream of aPKC, we assessed discs co-expressing *aPKC*^*act*^ and a moderate strength RNAi against *yki* (*ykiRNAi*). While *yki* knockdown under these conditions had a minimal effect on WT growth, it completely abrogated ectopic aPKC*-*driven *upd3.3LacZ* upregulation (Figure 5C; Figure 4—figure supplement 1E; Figure 4—figure supplement 2B). Similarly, depletion of *yki* suppressed the overgrowth of *scrib* tissue (Figure 4—figure supplement 3I–J). We then analyzed discs overexpressing constitutively active Yki (*Yki*^*act*^), which display massive overgrowth without affecting epithelial polarity (Dong et al., 2007; Oh and Irvine, 2008). *Upd3.3LacZ* expression was highly elevated in *Yki*^*act*^-expressing tissues (Figure 5D–E; Figure 4—figure supplement 2C), indicating that Yki can also be sufficient to activate the polarity-responsive enhancer.

### Coincident activation of *upd3.3* by aPKC and JNK

Though hyperactivation of either JNK or Yki through overexpression of activated proteins can drive *upd3.3* transcription, we found that only the highest levels of signaling could do so. For instance, neither *upd3*.3*lacZ* nor JAK/STAT signaling was active in hyperproliferating *hippo* (*hpo*) pathway mutant tumors (Figure 5—figure supplement 2C–F). Moreover, overexpression of either WT JNK kinase, or a membrane-targeted form of WT aPKC (aPKC^mild^), activated Mmp1 but does not cause substantial overgrowth; neither activates *upd3.3lacZ* (Figures 5F–G, 7J, Figure 5—figure supplement 3B)*.* Since loss of polarity activates aPKC and JNK signaling in parallel, we tested whether the two pathways converge upon the enhancer. Strikingly, coexpression of JNK kinase and aPKC^mild^ induced *upd3.3lacZ* upregulation (Figure 5H; Figure 4—figure supplement 2D), along with moderate overgrowth and polarity defects. These data support a model in which *upd3.3* works as a ‘coincidence detector’, responding to simultaneous aPKC-mediated Yki activation and JNK-dependent Fos activation upon polarity loss.

### Epigenetic regulation of polarity-responsive targets

**Figure 6.**
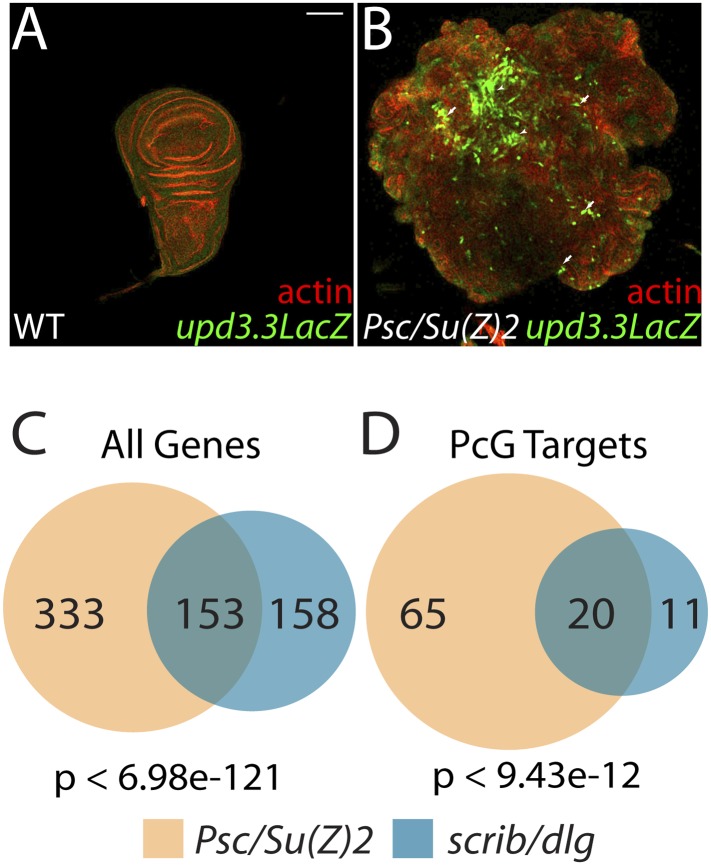
The Scrib module and PcGs regulate common targets. (**A** and **B**) Loss of the paralogous PcGs *Psc* and *Su(z)2* leads to activation of *upd3.3lacZ*, along with dramatic overgrowth and architecture defects. Activation is observed in areas of epithelial (arrows) and disrupted (arrowheads) organization. Comparison of all genes (**C**) and direct PcG targets (**D**) upregulated in *Psc/Su(z)2* and Scrib module mutant tissues reveals statistically significant overlaps. Scale bar: 100 μm. **DOI:** http://dx.doi.org/10.7554/eLife.03189.019

The above results suggest that transcription from enhancers like *upd3.3* is kept in check when either JNK or Yki are activated at physiological rather than manipulated experimental levels. We therefore investigated additional regulators of *upd* transcription. Our previous work identified the *upd* genes as targets of direct repression by the Polycomb Group (PcG), and showed that mutations in PcG can result in tumorous growth (Classen et al., 2009). These data suggest the hypothesis that epithelial polarity also acts through PcG to influence mitogenic gene expression. To test this hypothesis, we first asked whether PcG regulates the polarity-responsive enhancer. Imaginal discs mutant for the paralogous PcGs *Psc* and *Su(z)2* show dramatic overgrowth, in which apicobasal polarity is often intact (Classen et al., 2009). Strikingly, they also upregulated *upd3.3LacZ*, but not other *upd3LacZ* subfragments (Figure 6B and data not shown). This response is identical to that observed in polarity-deficient tissues.

**Figure 6—figure supplement 1.**
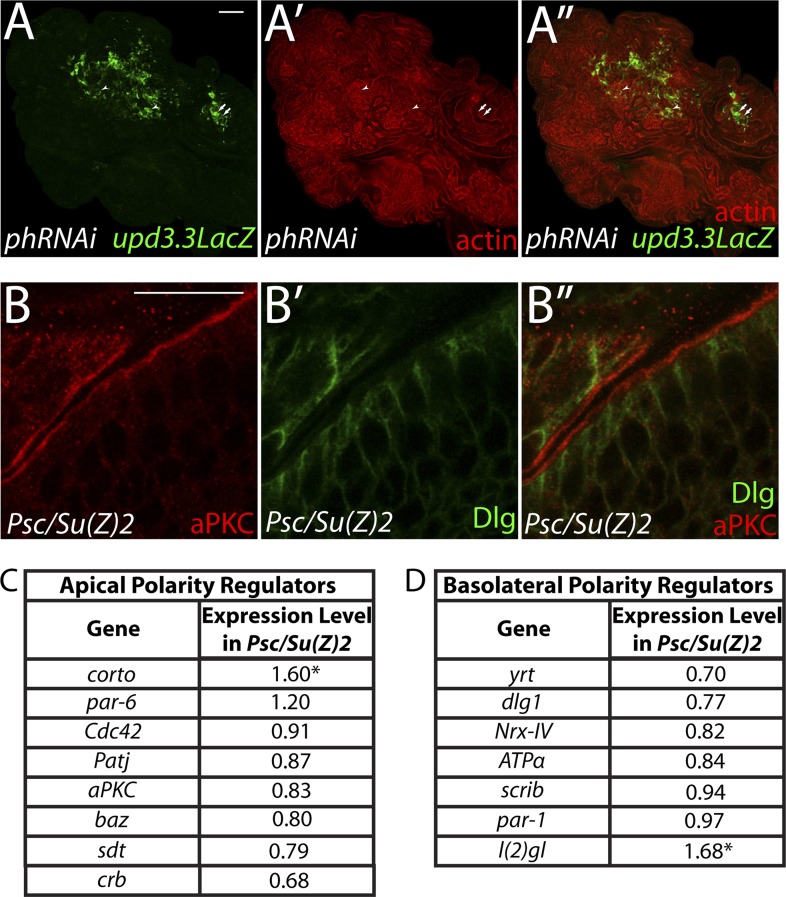
PcG depletion does not cause widespread loss of polarity. (**A**) Depletion of the paralogous PcGs *ph-p* and *ph-d* leads to overgrowth and *upd3.3LacZ* activation, including in areas with mild architecture defects. Arrows show areas of *upd3.3LacZ* expression in areas with epithelial organization; arrowheads indicate reporter activation in regions with disrupted architecture. (**B**) Regions of *Psc/Su(Z)2* mutant discs have normal polarity. (**C** and **D**) Most apical and basolateral regulatory genes are minimally changed in *Psc/Su(Z)2* tissue. Scale bars: **A**: 100 μm, **B**: 10 μm. **DOI:** http://dx.doi.org/10.7554/eLife.03189.020

The common response of *upd3* to polarity regulators and PcG could be a unique case, or alternatively could reflect a larger role for PcG in polarity-sensitive growth control. To determine if the Scrib module and PcGs co-regulate additional loci, we carried out a global transcriptional analysis of PcG mutant wing disc tumors (Supplementary file 3). Comparison of Scrib module and PcG mutant RNA-Seq datasets revealed that nearly half of the genes upregulated upon polarity loss are also upregulated in PcG mutant tissues, a highly significant enrichment (p < 6.98e-121, Figure 6C). This degree of similarity does not reflect a general overgrowth signature, as comparison with the transcriptome of *warts* tumors (Oh et al., 2013) gives a much less substantial overlap (Figure 5—figure supplement 2A). Further analysis of Scrib module transcriptomes revealed that nearly 25% of direct Pc-bound targets (Kwong et al., 2008) that are upregulated upon PcG loss are also upregulated in polarity-deficient tissues (Figure 6D). This strong enrichment supports a model whereby the Scrib module and PcG act in concert at certain common downstream genes.

### Polarity regulates PcG component transcription to modulate mitogenic gene expression

**Figure 7.**
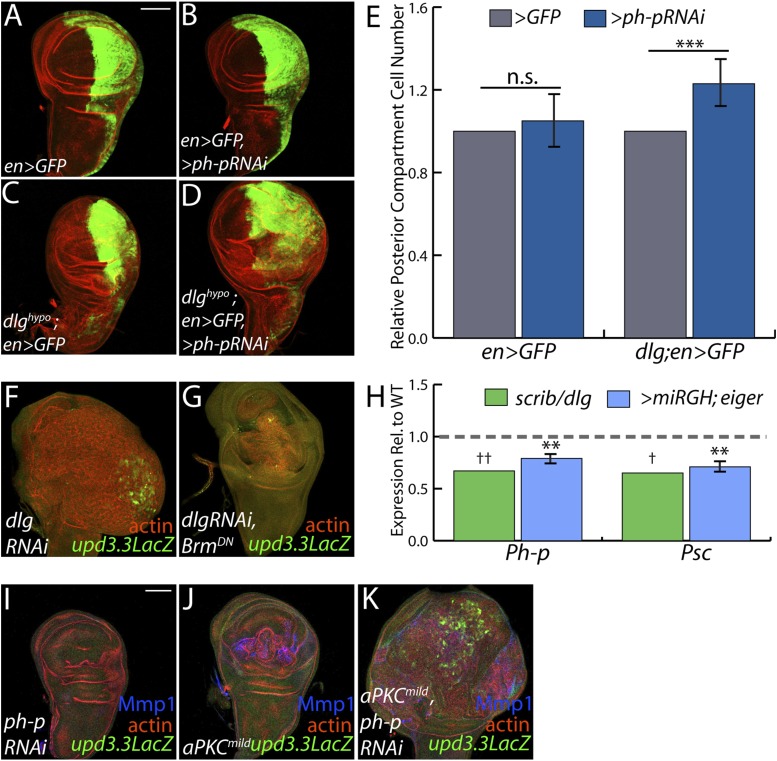
PcGs cooperate with Scrib module proteins to regulate growth. (**A**–**D**) Knockdown of *ph-p* has little effect on WT growth but increases the growth of *dlg*^*hypo*^ tissue. Quantification is in **E** (***p < 0.0001). (**F**–**G**) *Brm*^*DN*^ expression in *dlgRNAi* tissue decreases both *upd3.3LacZ* activation and overgrowth. (**H**) PcG components *ph-p* and *Psc* are downregulated in *scrib* and *dlg* mutant tissue (average in green), similar to levels observed upon JNK activation (blue). (**p < 0.005 ^✝^FDR < 0.05 in one genotype; ^✝✝^FDR < 0.05 in both genotypes) (**I**–**K**) Knockdown of *ph-p* or expression of a moderately active form of aPKC (*aPKC*^*mild*^) does not induce *upd3.3LacZ*, and *aPKC*^*mild*^ induces only slight overgrowth. However, co-expression of these transgenes leads to strong overgrowth and *upd3.3LacZ* expression. Scale bar: 100 μm. **DOI:** http://dx.doi.org/10.7554/eLife.03189.018

The above data are consistent with a scenario whereby polarity loss weakens PcG-mediated repression of select targets that promote tumorigenesis. An alternate possibility is that PcG mutant tissue itself is polarity-defective; however, it often maintains polarized organization including areas that upregulate *upd3.3lacZ*, it does not show transcriptional changes of polarity regulators, and unlike neoplastic tissue it is not suppressed by aPKC inhibition ([Classen et al., 2009], Figure 6—figure supplement 1, data not shown). To assess the functional significance of PcG in neoplastic tissues, we used the genetic interaction assay of Figure 2. Knockdown of the PcG gene *polyhomeotic-proximal* (*ph-p*) alone has no effect on growth of WT discs, due to the presence of its paralog *polyhomeotic-distal* (*ph-d*). However, when *ph-p* is knocked down in hypomorphic *dlg* discs, it significantly increased growth and cell proliferation (Figure 7A–E). Similar results were observed upon knockdown of a second PcG component, *Su(Z)2* (data not shown). If reduced PcG function contributes to overgrowth upon polarity loss, then preventing target derepression should suppress neoplastic growth. We inhibited Brahma (Brm), which suppresses PcG-mediated homeotic transformation and often opposes PcG activity at target genes (Tamkun et al., 1992). Expression of dominant-negative Brm reduced both the growth of *dlgRNAi*-expressing tissue and *upd3.3LacZ* expression (Figure 7F–G, Figure 4—figure supplement 2A). An analogous experiment with *scrib RNAi* could not be performed due to synthetic lethality with the *Brm-DN* transgene. These data support a role for epithelial polarity in promoting PcG-mediated repression of mitogenic target genes to suppress tumorigenesis.

The above analyses suggest diminished PcG activity in Scrib module mutant tissues, but do not point to a molecular mechanism. Intriguingly, using a wounding paradigm, Lee et al. found that JNK signaling can partially downregulate PcG expression, facilitating dedifferentiation and regeneration (Lee et al., 2005). Because JNK is activated upon polarity loss, we evaluated PcG transcript levels in Scrib module mutant tissues. Expression of the core PcG components *ph-p* and *Psc* is reduced in neoplastic tumors to an extent similar to that seen upon strong JNK activation (Figure 7H), suggesting that JNK signaling upon polarity loss compromises PcG function.

Finally, we tested whether compromised PcG function would promote polarity-responsive enhancer activation under moderate signaling conditions. Mild activation of aPKC drove polarity alterations and a limited degree of neoplasia, along with mild JNK signaling that can activate Mmp1; at these levels, both kinases together were incapable of activating *upd3.3* (Figure 7J). However, upon knockdown of *ph-p*, which does not activate JNK, mild *aPKC* signaling not only drove robust overgrowth but also *upd3.3LacZ* upregulation (Figure 7I–K, Figure 4—figure supplement 2D). From these data, we conclude that epithelial polarity normally suppresses neoplasia through PcG in cooperation with JNK and aPKC/Yki pathways.

## Discussion

Studies in vertebrate and invertebrate tissues have revealed intimate links between epithelial organization and the control of tumorous characteristics such as cell proliferation, differentiation, and motility. Here, we analyze both global RNA expression and a single polarity-responsive enhancer to delineate the signaling, transcriptional and epigenetic pathways linking epithelial organization to these diverse phenotypes. In polarity-deficient tissues, the simultaneous initiation of Fos-dependent transcription, aPKC-mediated Yki activation, and loss of PcG target repression leads to induction of a broad group of oncogenic factors, including the mitogenic JAK/STAT ligands. Our work provides insight into the logic, as well as the molecular mechanisms, by which polarity maintenance acts as a tumor-suppressive feature.

### Linking polarity to growth control

Our data build on those of others showing that JNK, aPKC and Yki are key players in fly neoplasia (Leong et al., 2009; Grzeschik et al., 2010; Robinson et al., 2010; Zhu et al., 2010; Doggett et al., 2011; Sun and Irvine, 2011; Verghese et al., 2012). By focusing on a single enhancer element of a gene involved in tumorous growth, we clarify the role of implicated regulating kinases and define how proliferation can be triggered by each pathway. Inhibition of Fos can suppress *upd3* upregulation and neoplasia, indicating that this transcription factor itself is the major target of JNK in this context. Yet a polarity-sensitive enhancer is not fully activated by JNK alone, even when apoptosis is blocked. aPKC is an additional regulator of this enhancer, and as previously suggested (Doggett et al., 2011), can activate Yki independent of, rather than through, JNK. Inhibiting either the JNK or Hpo pathways, including depletion of the downstream transcription factors, prevents expression of the polarity-sensitive enhancer; our analysis predicts that mutating transcription factor binding sites would give the same effect. Knockdown of *upd3* alone in neoplastic tumors does not prevent overgrowth (Figure 2—figure supplement 1); *upd1* and *upd2* are also regulated by JNK, Yki, and PcG (Pastor-Pareja et al., 2008; Classen et al., 2009; Jiang et al., 2009; Staley and Irvine, 2010; Wu et al., 2010) and may act through analogous enhancers to cooperatively drive tumor formation.

Loss of polarity thus induces two separate signaling pathways. An unknown mechanism triggers JNK to induce Fos-dependent transcription, while at the same time mispolarization of aPKC drives Yki-dependent transcription. Under mild signaling conditions, both pathways are required simultaneously to trigger enhancer expression or overgrowth, while inhibition of either is sufficient to suppress neoplasia. We suggest that polarity-responsive enhancers like *upd3.3* work as ‘coincidence detectors’ that during normal physiology require inputs from both JNK/Fos and aPKC/Yki. In this way, neither stress nor developmental growth signals alone run the risk of triggering malignant transformation. However, upon severe tissue damage that disrupts the epithelium, both stress and polarity signals are initiated to effect repair pathways (see below).

Our results also emphasize the unexpectedly central role of transcription in mediating cell polarity loss. Inhibition of Fos can revert not only growth defects but also polarity defects of neoplastic tumors. This surprising result suggests that polarity regulation by the Scribble module involves not only antagonistic interactions with the Par module at the cell cortex, but also an important transcriptional component that may be regulated similarly to the mitogenic *upd3* enhancer studied here. Nevertheless, activation of JNK, Yki, or both together is insufficient to elicit polarity defects (Figure 5—figure supplement 3), while aPKC activation alone is. Thus, aPKC must have additional effectors through which it regulates transformation; further analysis of the neoplastic transcriptome will shed light on this.

### Yki in neoplastic and hyperplastic growth

Yki is clearly a major regulator of neoplastic transformation, providing a link between the primary *Drosophila* TSG pathways (Grzeschik et al., 2010; Robinson et al., 2010; Chen et al., 2012; Verghese et al., 2012). However, our transcriptional data highlight a major puzzle. Many Hpo pathway targets, including direct growth regulators such as *cycE*, *diap1*, and *Myc*, are expressed at near-normal levels in Scrib module mutants, and comparison of Scrib module and Hpo mutant transcriptomes reveals limited overlap (Figure 5—figure supplement 2A–B). If Yki is activated in both types of tumorous tissue, why do they behave so differently? Our data help to rule out several models for altered Yki target selection. It is unlikely to be driven by simultaneous activation of JNK upon polarity loss, since co-activation of Yki and JNK does not recapitulate neoplastic growth phenotypes (Figure 5—figure supplement 3). It is also unlikely to be explained by a model in which Yki activation through aPKC differs from Yki activation through canonical Hpo pathway regulators, since a transgenic 3.5 kb *diap1* fragment is strongly upregulated in neoplastic tissue, paralleling upregulation of a minimal Yki-responsive element (Figure 5—figure supplement 2G–J). Interestingly, an enhancer trap inserted at the same 3.5 kb sequence in the endogenous *diap1* locus (Zhang et al., 2008) is only slightly upregulated by comparison (Figure 5—figure supplement 2K–L), hinting that the native chromatin environment at certain Yki targets might influence target response.

### Polarity and epigenetic regulation

Our data point to PcG as a new player in the transcriptional response to polarity loss. Three pieces of evidence support a close relationship between the Scrib module and PcGs: (1) their related mutant phenotypes, (2) the extensive and highly significant overlap of their mutant gene expression profiles, and (3) the sensitivity of Scrib module mutant overgrowth to changes in PcG activity. However, since canonical PcG targets including Hox genes are not upregulated in neoplastic tissues (Supplementary files 1–2), and overall Histone H3K27me3 levels are not altered (data not shown), the data rule out a global inactivation of PcG. Instead, they suggest that decreased PcG-mediated repression ‘primes’ select targets for activation by polarity-responsive effector pathways. Mild activation of either JNK or aPKC alone is insufficient to stimulate enhancers such as *upd3.3*. However, at these targets, reduced PcG activity upon Scrib module loss synergizes with JNK and aPKC signaling, perhaps by providing a permissive chromatin environment for Fos- and Yki-stimulated transcription. More generally, the link to epigenetic regulators that control many targets provides a mechanism by which loss of a single polarity regulator can induce the widespread transcriptional changes that drive the multifaceted neoplastic phenotype.

### Tumor characteristics revealed by the neoplastic transcriptome

Our primary analysis focuses on overgrowth, but the transcriptome identifies further features of human cancer found in neoplastic *Drosophila* cells. In addition to oxidative stress, fly homologs of metabolic genes that fuel human cancer growth are elevated, including fatty acid synthase (FASN) which facilitates de novo lipogenesis, and LDH which promotes aerobic glycolysis in the Warburg effect (Cairns et al., 2011; Baenke et al., 2013; Gorrini et al., 2013). However, glycolytic enzyme transcription in fly neoplastic tumors remains relatively unchanged, suggesting that metabolic changes may be more complex*.* Dedifferentiation is considered another key feature of human tumor malignancy (Friedmann-Morvinski and Verma, 2014), and the major signature evident from genes downregulated in neoplastic tissues reflects a failure to differentiate. Khan et al. recently reported that forcing differentiation can cause elimination of neoplastic clones (Khan et al., 2013); by contrast, our experiments show that restoring expression of the wing-fate regulator Vg suppresses tumorous overgrowth without inducing cell death. Thus, promoting tissue differentiation may be a tumor suppressive function of epithelial organization.

Why might loss of polarity drive this particular constellation of events that result in tumorous overgrowth? Our global analysis reveals that apicobasal polarity disruption elicits responses with striking parallels to those seen in epithelial wounds in both *Drosophila* and humans (Schäfer and Werner, 2008; Lee and Miura, 2014). These parallels, which are both thematic and extend to regulation of specific genes, include activation of stress signaling, reactive oxygen species production, upregulation of matrix remodeling enzymes, de-differentiation, recruitment of immune cells, and transcription of growth-promoting cytokines that stimulate cell proliferation. Intriguingly, several upregulated neoplastic effectors that contain conserved AP-1 and Sd binding sites are also upregulated during wound-healing (Pastor-Pareja et al., 2008; Wu et al., 2009; Garelli et al., 2012; Patterson et al., 2013) (Figure 3—figure supplement 2). An attractive model is that linking transcriptional control of such targets to polarity regulators, via polarity-regulated aPKC, cell architecture-regulated Yki and stress-regulated JNK activity on both downstream transcription factors and PcG epigenetic regulators, allows the tissue to connect disturbances in its integrity to the activation of broad gene expression programs that promote repair. Following tissue damage, restoration of tissue architecture and integrity would abrogate wound-response signals. In contrast, in polarity-deficient tissues, architecture can never be restored, and these pro-growth, de-differentiation cues remain active, leading to the formation of malignant tumors that kill the organism. Our data linking apicobasal polarity to neoplastic gene expression thus suggest an evolutionarily ancient genesis for cancers as ‘wounds that never heal’ (Dvorak, 1986).

## Materials and methods

### *Drosophila* genetics

The following alleles were used in this study: *white [1118]* (WT), *dlg [40-2]*, *dlg [hf321]* (*dlg*^*hypo*^) *scrib [1]*, *hep [r75]* (JNKK), *Psc/Su(Z)2 [XL26]* (Li et al., 2010), *yki*^*B5*^. The following additional strains were used: *engrailed GAL4*, *UAS-GFP*, *ms1096 GAL4*, *eyFLP*; *act>>GAL4*, *UAS-GFP*, *10XStat92E-GFP*, *upd3.1LacZ*, *upd3.2LacZ*, and *upd3.3LacZ* (Jiang et al., 2011), *th*^*j5c8*^ (Ryoo et al., 2002), *diap1-GFP3.5* (Zhang et al., 2008), *HREX-GFP* (Wu et al., 2008), *UAS-Socs36E*, *UAS-Dome*^*∆cyt*^ (*UAS-Dome*^*DN*^), *UAS-Bsk*^*K53R*^ (*UAS-JNK*^*DN*^), *UAS-fos*^*panAla*^ (*UAS-Fos*^*DN*^), *UAS-miRNA*^*reapergrimhid*^ (*UAS-miRGH*) (Siegrist et al., 2010), *UAS-GFP*, *UAS-hippo*, *UAS-eiger*, *UAS-aPKC*^*ΔN*^ (*UAS-aPKC*^*act*^), *UAS-yki*^*S168A*^ (*UAS-yki*^*act*^), *UAS-Brm*^*K804R*^ (*UAS-brm*^*DN*^), *UAS-aPKC*^*CAAX*^ (*UAS-aPKC*^*mild*^), *UAS-Sod2*, *UAS-Catalase*, *UAS-Ey*, *UAS-vg*, and *UAS-hep*^*WT*^ (*UAS-JNKK*^*WT*^), *AP-1-GFP*, *ImpL2-GFP*, *dilp8-GFP*, *EcadRNAi*. *UAS-aPKC*^*CAAX*^ *UAS-Par6* was a kind gift from T Harris. *UAS-dlgRNAi* (*39035*), *UAS-dlgRNAi* (*34854*) were obtained from the Bloomington Stock Center; *UAS-yki RNAi* (*104523*), *UAS-ph-p RNAi* (*10679*), *UAS-ph RNAi* (*50028*), and *UAS-Su(Z)2 RNAi* (*100096*) were obtained from the Vienna Drosophila RNAi Center. Unless otherwise noted, all transgenes were driven in the wing pouch by *ms1096-GAL4*. WT controls were outcrosses to *w*. Crosses were reared at 25°C, except for the crosses to assess *upd3.3LacZ* expression in *scribIR* and *scribIR;Bsk*^*DN*^ tissue, which were raised at 29°C.

### Immunofluorescence and microscopy

Imaginal discs were fixed and stained (Bilder et al., 2000) with TRITC-phalloidin (Sigma-Adrich, St. Louis, MO) and primary antibodies against the following antigens: β-gal (Abcam, San Francisco, CA), Mmp1, Dlg, Scrib (all from Developmental Studies Hybridoma Bank, Iowa City, IA) and aPKC (Santa Cruz Biotechnology, Santa Cruz, CA). DAPI (Molecular Probes, Eugene, OR) was used to visualize nuclei. Secondary antibodies were from Invitrogen (Carlsbad, CA). DHE staining was performed on live tissue as previously described (Owusu-Ansah et al., 2008). Mutant and WT discs were stained in the same tube and imaged under identical confocal settings. Images are single cross-sections obtained on either a Leica TCS or a Zeiss LSM 700 and processed with Adobe Photoshop CS2 12.0.1. Bgal staining was quantified as the percentage of pixels above background and normalized to WT levels.

### mRNA purification, sequencing, and data analysis

At least 50 wing imaginal discs were dissected from *white*^*1118*^, *scrib*^*1*^, and *dlg*^*40-2*^*/Y* larvae for each biological replicate, and at least two biological replicates were sequenced per genotype. *Psc/Su(Z)2 [XL26] FRT42* and control isogenized *FRT42* wing discs were generated using *UbxFLP; cell-lethal* as described (Newsome et al., 2000). Control tissue was isolated 5–6 days after egg lay (AEL), while tumorous discs was isolated 7–8 days AEL to account for the developmental delay of tumor-bearing larvae. Poly-A transcripts were purified via two rounds of extraction using the MicroPolyAPurist kit (Ambion, Austin, TX). mRNA was subsequently prepared for sequencing (Dalton et al., 2013).

Libraries were sequenced by 50-bp single-end reads on either the GAIIX Genome Analyzer or HighSeq2000 platform (Illumina, San Diego, CA). Reads were aligned to the *Drosophila melanogaster* reference genome (version 5.43) using TopHat run under default parameters (Langmead et al., 2009). The number of reads from each replicate falling on each exon was counted using HTSeq (Anders et al., 2015) in the UNION mode, and the differential expression levels across all of samples were calculated using DESeq (Anders and Huber, 2010). Normalized value for gene expression is reported in a single ‘reads per kilobase gene length per million total reads’ (RPKM) value for each gene. Supplementary file 4 contains the sequencing and mapping statistics for each replicate, and Supplementary file 5 contains the number of differentially expressed genes for each genotype.

For binding profile comparison, genes associated with Pc binding (peak_hit, peak_near, gray_hit, gray_near) in thoracic imaginal discs (Kwong et al., 2008) were defined as PcG targets. Genes upregulated at least twofold and having an RPKM value of at least 10.0 in *wts* mutant tissue were used to assess the overlap of the Scrib module and Hippo pathway mutant transcriptome profiles (Oh et al., 2013). p-values for significance of overlap of transcriptome profiles was found using hypergeometric probability. Gene Ontology analysis was performed using GoStat (Beissbarth and Speed, 2004).

### qRT-PCR

Total RNA was isolated from at least 20 wing discs co-expressing *eiger* and *miRGH* with *ms1096 GAL4*, along with outcrossed controls, using the RNeasy Mini Kit (Qiagen, Valencia, CA), and cDNA was generated from 500 μg of RNA using Superscript II Reverse Transcriptase (Life Technologies, Carlsbad, CA). Quantitative real-time PCR was performed using SYBR GreenER qPCR SuperMix (Invitrogen, Carlsbad, CA) on a StepOnePlus ABI Machine (Applied Biosystems, Foster City, CA). Relative gene expression levels were quantified using the ΔΔC_T_ method, after normalization to three endogenous control genes (*GAPDH*, *CG12703*, *Cp1*). Average fold expression of at least four biological replicates is shown. Primer sequences are listed in Supplementary file 6.

### Cloning *upd3LacZ*

Genomic DNA was isolated from adult flies using standard procedures. The *upd3* fragment was amplified using Phusion High Fidelity Polymerase (NEB) and the following primers: 5′-GGTGGTACCTCGTACAATGGTTTAAAAATAGCTCGGCCAA-3′ and 5′-GGAAGGCCTCTCCTACACATCGAGCAGCATGGTCAACGAA-3′. The 3-kb fragment was ligated into a pH-Pelican-attB vector and sequence was confirmed. Transformation into the *attP2* landing site was performed by BestGene, Inc (Chino Hills, CA).

### Fluorescence activated cell Sorting analysis

At least 10 wing discs were dissected and disassociated as described (de la Cruz and Edgar, 2008). Cells were counted using an EPICS XL flow cytometer (Beckman–Coulter, Brea, CA). GFP+ and GFP− gates were generated based on a *white*^*1118*^ negative control sample. To calculate Relative Posterior Compartment Size, the number of GFP+ cells was divided by the total number of live cells and normalized to control discs. A two-tailed Student's *t*-test was used to calculate the p-values based on at least three biological replicates for each genotype.

## Additional files

Supplementary file 1. Transcriptome Analysis of *scrib* Tissue. Differential expression analysis of *scrib* versus *white* RNA-Seq data by DESeq. Each column contains the following information: Flybase ID- Flybase Gene Identifier; Gene Name- Name of each gene; baseMean_allconditions- Average normalized read count for that gene, across all samples, baseMean_white- Normalized read count for that gene in *white* tissue; baseMean_scribble- Normalized read count for that gene in *scrib* tissue; foldChange- Change of the gene in *scribble*, relative to *white* tissue; foldChangelog2- Logarithm to base 2 of the fold change; pval- p-value for the statistical significance of the fold change; padj- p-value adjusted for multiple testing with the Benjamini-Hochberg procedure, which controls for false discovery rate (FDR). **DOI:** http://dx.doi.org/10.7554/eLife.03189.021

Supplementary file 2. Transcriptome Analysis of *dlg* Tissue. Differential expression analysis of *dlg* versus *white* RNA-Seq data by DESeq. Each column contains the following information: Flybase ID- Flybase Gene Identifier; Gene Name- Name of each gene; baseMean_allconditions- Average normalized read count for that gene, across all samples, baseMean_white- Normalized read count for that gene in *white* tissue; baseMean_discslarge- Normalized read count for that gene in *dlg* tissue; foldChange- Change of the gene in *dlg*, relative to *white* tissue; foldChangelog2- Logarithm to base 2 of the fold change; pval- p-value for the statistical significance of the fold change; padj- p-value adjusted for multiple testing with the Benjamini-Hochberg procedure, which controls for false discovery rate (FDR). **DOI:** http://dx.doi.org/10.7554/eLife.03189.022

Supplementary file 3. Transcriptome Analysis of *Psc/Su(Z)2* Tissue. Differential expression analysis of *scrib* versus *white* RNA-Seq data by DESeq. Each column contains the following information: Flybase ID- Flybase Gene Identifier; Gene Name- Name of each gene; baseMean_allconditions- Average normalized read count for that gene, across all samples, baseMean_iso- Normalized read count for that gene in *iso42* tissue; baseMean_PscSuZ2- Normalized read count for that gene in *Psc/Su(Z)2* tissue; foldChange- Change of the gene in *scribble*, relative to *white* tissue; foldChangelog2- Logarithm to base 2 of the fold change; pval- p-value for the statistical significance of the fold change; padj- p-value adjusted for multiple testing with the Benjamini-Hochberg procedure, which controls for false discovery rate (FDR). **DOI:** http://dx.doi.org/10.7554/eLife.03189.023

Supplementary file 4. RNA-Seq alignment statistics. Table of combined number of 50-bp single-end sequencing reads for each sequencing replicate. Reads were considered ‘non-aligned’ if they had >2 mismatches relative to the reference genome, and ‘low complexity’ reads had multiple matches within the genome, reflecting sequencing reads from repeated DNA elements. Percentages listed refer to the number of reads for each category relative to the total number of reads. **DOI:** http://dx.doi.org/10.7554/eLife.03189.024

Supplementary file 5. Contains the number of differentially expressed genes for each genotype. **DOI:** http://dx.doi.org/10.7554/eLife.03189.025

Supplementary file 6. Contains the primer sequences used for quantitative PCR in Figure 7. **DOI:** http://dx.doi.org/10.7554/eLife.03189.026
